# The association between age of onset of opioid use and comorbidity among opioid dependent patients receiving methadone maintenance therapy

**DOI:** 10.1186/s13722-017-0074-0

**Published:** 2017-03-28

**Authors:** Leen Naji, Brittany Burns Dennis, Monica Bawor, Michael Varenbut, Jeff Daiter, Carolyn Plater, Guillaume Pare, David C. Marsh, Andrew Worster, Dipika Desai, James MacKillop, Lehana Thabane, Zainab Samaan

**Affiliations:** 1 Michael Degroote School of Medicine, McMaster University, Hamilton, Canada; 2 St. George’s University of London, London, UK; 3 Department of Clinical Epidemiology and Biostatistics, McMaster University, Hamilton, Canada; 4 Canadian Addiction Treatment Centres, Richmond Hill, Canada; 5 Northern Ontario School of Medicine, Sudbury, ON Canada; 6 Department of Medicine, Hamilton General Hospital, Hamilton, Canada; 7 Population Genomic Program, Chanchalani Research Centre, McMaster University, Hamilton, Canada; 8 Departments of Pediatrics and Anesthesia, McMaster University, Hamilton, Canada; 9 Centre for Evaluation of Medicine, St Joseph’s Healthcare, Hamilton, Canada; 10 Biostatistics Unit, Father Sean O’Sullivan Research Centre, St Joseph’s Healthcare, Hamilton, Canada; 11 Peter Boris Centre for Addictions Research, Hamilton, Canada; 12 Department of Psychiatry and Behavioural Neuroscience, McMaster University, 100 West 5th Street, Hamilton, ON L8N 3K7 Canada

**Keywords:** Opioid use disorder, Risk prediction, Substance use, Methadone, Opioid substitution treatment

## Abstract

**Background:**

Opioid use disorder (OUD) affects approximately 21.9 million people worldwide. This study aims to determine the association between age of onset of opioid use and comorbid disorders, both physical and psychiatric, in patients receiving methadone maintenance treatment (MMT) for OUD. Understanding this association may inform clinical practice about important prognostic factors of patients on MMT, enabling clinicians to identify high-risk patients.

**Methods:**

This study includes data collected between June 2011 and August 2016 for the Genetics of Opioid Addiction research collaborative between McMaster University and the Canadian Addiction Treatment Centers. All patients were interviewed by trained health professionals using the Mini-International Neuropsychiatric Interview and case report forms. Physical comorbidities were verified using patients’ electronic medical records. A multi-variable logistic regression model was constructed to determine the strength of the association between age of onset of opioid use and the presence of physical or psychiatric comorbidity while adjusting for current age, sex, body mass index, methadone dose and smoking status.

**Results:**

Data from 627 MMT patients with a mean age of 38.8 years (SD = 11.07) were analyzed. Individuals with an age of onset of opioid use younger than 18 years were found to be at higher odds for having a physical or psychiatric comorbid disorder compared to individuals with an age of onset of opioid use of 31 years or older (odds ratio 2.94, 95% confidence interval 1.20, 7.19, *p* = 0.02). A significant association was not found between the risk of having a comorbidity and an age of onset of opioid use between 18 and 25 years or 26 and 30 years, compared to an age of onset of opioid use of 31 years or older.

**Conclusion:**

Our study demonstrates that the younger one begins to use opioids, the greater their chance of having a physical or psychiatric co-morbidity. Understanding the risk posed by an earlier onset of opioid use for the later development of comorbid disorders informs clinical practice about important prognostic predictors and aids in the identification of high-risk patients.

## Background

Opioid use disorder (OUD) is a serious health concern associated with significant morbidity and mortality [1]. It is the second most prevalent illicit drug use disorder, affecting approximately 21.9 million people worldwide [1]. Despite evidence that the prevalence of pain has remained unchanged, the rate of opioid prescriptions has nearly quadrupled since 1999 [2]. Estimates based on The National Survey on Drug Use and Health indicate that 12.5 million people in the United States alone misused opioids in 2015, in addition to the 0.8 million people who misused heroin that year [3].

Previous research demonstrates a potential predictive association between the use of opioids and the presence of comorbidities, likely secondary to a variety of factors including chronic intoxication, poor hygiene, life style habits and risk-taking behavior [4–17]. For instance, the prevalence of human immunodeficiency virus (HIV) is found to be higher among opioid users compared to non-users, as is the prevalence of infections such as hepatitis and tuberculosis [16, 18, 19]. Several studies have also linked opioid use to serious cardiac abnormalities, such as infective endocarditis, and a higher rate of epileptic seizures [5, 6, 9, 13]. Additionally, opioid use has been associated with chronic pain, liver disease and mental health comorbidities, such as depression and anxiety disorders [4, 10, 12]. While these studies do not establish causation, potential pathophysiological processes contributing to the development of these comorbidities may be hypothesized. For instance, while infectious diseases may be associated with injecting drug use and risk-taking behavior, the neurological abnormalities are likely due to the neurotoxic effects of drug use leading to cerebral lesions [15].

Understanding the association between age of onset of opioid use and the presence of physical or psychiatric comorbidity has seldom been the primary focus of previous studies. While addiction medicine clinicians may argue that pretreatment opioid use history seems logically inherent as a predictor for future health complications, there is a paucity of well-powered studies to support such claims [5, 11, 15, 17, 20]. The rare times this issue is addressed in the literature, the primary focus is often the correlation between long substance use history and neurological deficits or infectious diseases. For instance, studies have demonstrated neurological abnormalities such as epilepsy, polyneuropathy, and pressure palsy increased with the duration of opioid use disorder [15]. Additionally, some studies have found an association between the duration of heroin use and the presence of comorbidities, including HIV and hepatitis C [17, 20].

Nevertheless, no study to date has addressed nor quantified the risk between age of onset of opioid use and the presence of physical or psychiatric comorbidities. Thus, the purpose of this study is to investigate this association using data from an on-going prospective cohort study. Understanding the association between the age of onset of opioid use and the presence of comorbid disorders informs clinical practice about important prognostic factors, enabling clinicians to identify high-risk patients. The opportunity to identify these patients would allow us to monitor them more closely, provide more intensive treatment strategies as well as preventative measures to reduce the risk of them developing a disease and/or progressing in their course of illness. These findings may also help to educate the public on the risks of opioid use above and beyond the direct effects of drug use, as well as identify modifiable factors that may increase the future risk of comorbidity in patients with OUD.

## Methods

**Fig. 1:**
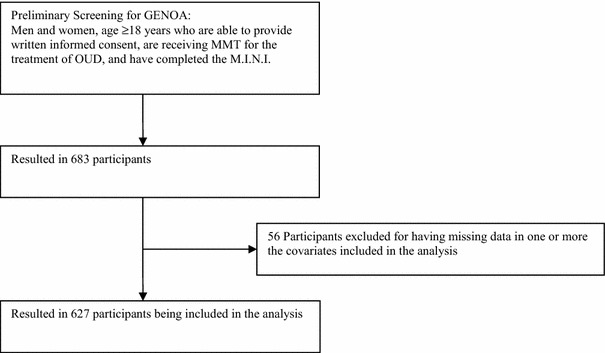
GENOA: eligibility screening and inclusion flow diagram for participant selection

This study was approved by the Hamilton Integrated Research Ethics Board (11-056). We used data collected primarily for the Genetics of Opioid Addiction (GENOA), a research collaborative between the Canadian Addiction Treatment Center (CATC, formerly known as the Ontario Addition Treatment Centre and home to the largest network of methadone clinics in North America) and McMaster University [21]. CATC clinical sites follow the same treatment strategy and are managed centrally. In order to be included in our study, participants had to be 18 years of age or older, had to provide written informed consent, and had to have been receiving methadone maintenance treatment (MMT) for OUD as defined in the Fifth Edition of the diagnostic and statistical manual of mental disorders (DSM-5) [22]. All study participants were interviewed by trained health care professionals between June 2011 and August 2016. Previously piloted case report forms were used to obtain patient data, and physical comorbidities were confirmed using patients’ electronic medical records [21]. To be included, participants must have also completed the Mini-International Neuropsychiatric Interview (M.I.N.I.) at the time of inclusion into the study, which was used to determine psychiatric comorbidities [23]. For this study, 683 patients met the eligibility criteria for inclusion (Fig. 1).

Our primary outcome was the composite of a physical or psychiatric co-morbid disorder. Physical comorbidities were identified through self-report and patients’ electronic medical records, and included HIV, hepatitis, liver disease, chronic pain and epilepsy, among others. Psychiatric diagnoses were ascertained based on the M.I.N.I. Patients were also required to complete a baseline questionnaire, providing details about their age of onset of opioid use, the duration of time they have been receiving MMT and their current methadone dose, prescribed and non-prescribed medications, as well as illicit substance use [21].

### Statistical analysis

We used descriptive statistics to summarize the participants’ demographic and baseline characteristics. We expressed continuous variables using mean [standard deviation (SD)] and categorical variables using percent. We performed the primary analysis by regressing the presence of a physical or psychiatric comorbidity, as composite outcome, onto the age of onset of opioid use after categorizing age of onset of opioid use into four categories: Children: 17 years and younger (n = 119), youth: 18–25 years of age (n = 244), young adults: 26–30 years of age (n = 97), and 31 years of age and older (n = 167). We conducted the regression such that each category is compared to the reference group of 31 years of age and older. We adjusted for important potential cofounders, specifically current age, sex, methadone dose, BMI and smoking status. Older age is known to be associated with more comorbidities [24]. Additionally, the prevalence of many psychiatric disorders such as depression and physical comorbidities such as chronic pain are more prevalent in women than men [25–31]. High doses of methadone are associated with prolonged QTc intervals and arrhythmias such as Torsades de pointes [32, 33]. Smokers and obese individuals are also at heightened risk for numerous serious comorbidities, including coronary artery disease and strokes [34, 35].

We performed secondary analyses investigating the association between the age of onset of opioid use and the presence of physical or psychiatric comorbidity, separately, while still adjusting for the aforementioned covariates as in the primary analysis.

We employed Stata 13 to complete the logistic regression analyses [36]. All study data have been quality checked and entered into Research Electronic Data Capture (RedCap) database at the Population Genomics Program, Chanchlani Research Centre, McMaster University server [37].

## Results

### Participants’ characteristics

Of the 683 participants eligible for inclusion into our study, those with missing values for one or more of the covariates analyzed were dropped from the analysis (n = 56). Therefore, we analyzed data from 627 subjects. Please refer to Fig. 1 for the participants’ inclusion diagram. The mean current age of participants was 38.8 years (SD = 11.1), more than half of whom were males (54.9%) (Table 1). The mean age of onset of opioid use was 25.6 years (SD = 8.8). The majority of participants (86.3%) had at least one comorbid physical or psychiatric disorder. Most (68.3%) participants were found to have at least one comorbid physical disorder, with chronic pain and hepatitis being the most common comorbidities (40.2 and 22.5% of participants, respectively). Additionally, 57.7% of participants were diagnosed with a psychiatric disorder, the majority of which constituted an anxiety disorder (33.8% of participants).

**Table 1:** Baseline participant characteristics (total N = 627)

	Mean (SD)	Median (IQR)
Participant characteristic		
Current age (years)	38.8 (11.1)	37.0 (19.0)
Methadone dose (mg/day)	77.24 (46.10)	70.00 (55.00)
Age of onset of opioid use (years)	25.6 (8.8)	24.0 (13.0)
BMI (kg/m^2^)	27.92 (8.88)	26.03 (7.76)

	Percentage of total (N)
Female	45.1 (283)
Current smoking	84.5 (530)
Participants with ≥1 comorbidity	86.3 (541)
Participants with physical comorbidity^a^	68.3 (428)
Participants with psychiatric comorbidity^b^	57.7 (362)
Age of onset of opioid use categories	
17 years and younger	19.0 (119)
18–25 years	38.9 (244)
26–30 years	15.5 (97)
31 years and older	26.6 (167)

^a^The physical comorbidities comprising the composite outcome included chronic pain (n = 252), epilepsy (n = 16), hepatitis (n = 141), liver disease (n = 31), HIV (n = 3), pulmonary disorders such as asthma and chronic obstructive pulmonary disorder (n = 52), diabetes mellitus type I or type II (n = 33), cardiovascular history including hypertension or cardiomyopathy (n = 33), arthritis (n = 25), inflammatory bowel disease (n = 6), endocrine disorders including hypothyroidism and hyperthyroidism (n = 15), cancer (n = 6), renal disorders (n = 5), neurological disorders including neuropathies and multiple sclerosis (n = 20), endometriosis (n = 7), dermatological disorders including eczema and psoriasis (n = 2), inactive TB (n = 2), and syphilis (n = 1)

^b^The psychiatric comorbidities comprising the composite outcome included mood disorders such as depression and mania (n = 87), anxiety disorders such as generalized anxiety and obsessive compulsive disorder (n = 212), substance use disorders other than opioids (n = 83), psychotic disorders (n = 24), eating disorders (n = 4), antisocial personality disorder (n = 144)

### Primary outcome

We determined the strength of the association between age of onset of opioid use and the presence of physical or psychiatric comorbidity using a multi-variable logistic regression model. A summary of our results is provided in Table 2. Younger age of onset was significantly correlated to the presence of a physical or psychiatric comorbidity, when adjusting for current age, sex, BMI, methadone dose and smoking status (Table 2). These results indicate that the risk of comorbidity is highest in those who started using opioids before 18 years of age, as they were almost three-fold more likely of to have a physical or psychiatric comorbidity compared to those who began using opioids at the age of 31 and older (odds ratio (OR) 2.94, 95% confidence interval (CI) 1.20, 7.19, *p* = 0.02). As expected, current age was also found to be significantly associated with physical or psychiatric comorbidity, implying that older individuals are at higher risk for the presence of a comorbidity (OR 1.05, 95% CI 1.02, 1.08, *p* < 0.01). Finally, women were more likely to have a physical or psychiatric comorbidity than men (OR 1.70, 95% CI 1.04, 2.75, *p* = 0.03).

**Table 2:** Multivariable logistic regression analysis: the presence of physical or psychiatric comorbidity (N = 627)

	Odds ratio, (95% confidence interval)	*p* value
Covariates		
Current age (years)	1.05 (1.02, 1.08)	<0.01*
Sex	1.70 (1.04, 2.75)	0.03
BMI	1.02 (0.99, 1.06)	0.19
Methadone dose (mg/day)	1.00 (1.00, 1.01)	0.77
Smoking status	1.56 (0.85, 2.88)	0.15
Age of onset of opioid use categories (in reference to 31 years and older)		
17 years old and younger	2.94 (1.20, 7.19)	0.02
18–25 years	1.71 (0.83, 3.51)	0.14
26–30 years	2.05 (0.89, 4.74)	0.09

56 eligible participants were excluded from the multi-variable regression analysis for having missing values for one or more of the covariates of interest. As such, 167, 97, 244 and 119 patients from the 31 years and older, 26–30 years, 18–25 years, and 17 years and younger subgroups were included in the analysis, respectively

* <0.01 = 0.002

Of note, our secondary analyses revealed that an age of onset of opioid use of younger than 18 years of age was significantly associated with an increased risk of having both a physical (OR 2.39, 95% CI 1.18, 4.85, *p* = 0.02) and psychiatric (OR 1.97, 95% CI 1.14, 3.40, *p* = 0.02) comorbidity, compared to those who began using opioids after the age of 30.

## Discussion

Our study revealed that within a sample of patients receiving MMT for OUD, there exists a significant association between the age of onset of opioid use and the presence of one or more psychiatric or physical comorbidity, including but not limited to anxiety disorder, chronic pain, liver disease, epilepsy, HIV and hepatitis. In other words, our study demonstrates that within this population, the younger one begins to use opioids, the greater their chance of having a physical or psychiatric co-morbidity.

If an earlier age of onset of opioid use is assumed to implicate a longer duration of substance use, the findings of our present study would be in accordance with the limited available literature investigating the association between duration of opioid use and the presence of a physical comorbidity [5, 11, 15, 17]. These investigations have found a positive association between duration of opioid use and the presence of neurological abnormalities or an HCV positive status [5, 11, 15, 17].

Additionally, our study is the first to quantify the association between the odds of having comorbidity and the age of onset of opioid use. Our results demonstrate that an age of onset of opioid use younger than 18 years of age is significantly associated with an increased likelihood of having a psychiatric comorbidity, compared to an age of onset beyond 30. Though we cannot claim causality due to the cross-sectional design of this study, the association between psychiatric comorbidity and a younger age of onset of opioid use may be explained by the fact that adolescence represents a period of time in which the brain is subject to critical neurochemical and functional changes, as well as maturing behavior and cognition [38]. Psychosocial and environmental factors, including drug use, are thought to interfere with this stage of development and may ultimately impact mental health [39]. Additionally, there exists a body of evidence that supports the presence of a positive correlation between drug use, including opioids, and psychiatric comorbidities other than substance use disorder [40–42].

Moreover, our study found that women with OUD were more likely than men to have a physical or psychiatric comorbidity. This is consistent with previously published literature which has found that women are more severely affected by the deleterious effects of drug use on physical and mental health [26, 29]. This may be explained by the fact many psychiatric disorders, including major depression and generalized anxiety disorder, are more prevalent in women than men in the general population [25, 27, 28]. As well, a study by Grella et al. [28] has found that this difference in prevalence is accentuated in patients with OUD. Moreover, chronic pain, which is the most prevalent physical comorbidity included in our composite outcome, is also more commonly found in women than in men in the general population [30, 31].

This study adds to the field of addiction by addressing age of onset of opioid use as a risk factor for concurrent physical ailments and psychiatric disorders in patients with OUD on MMT. Several factors render our study unique amongst the available literature on this topic, allowing us to draw more reliable conclusions from our findings. This study included a large sample size of a clinical population receiving standardized MMT across multiple different sites. Second, our investigation obtained an older sample of participants, where the mean age of participants was 38.8 years. This is in contrast to the available literature, which primarily focuses on opioid users in their mid-twenties [5, 11, 14, 15, 17]. Studying an older age group provides us with the opportunity to gain a better understanding of the risks associated with a longer duration of opioid use, as well as a better chance of identifying physical and psychiatric comorbidities that may not be captured in a younger cohort.

### Limitations

There are specific limitations of studying OUD, one being the multifaceted nature of addiction itself. There are numerous ways in which confounding can inevitably influence the results obtained during this study. OUD involves a complex network of variables all of which unavoidably modify individual patient health outcomes. It is important to note that this study has taken time to outline the most important confounders and adjust for them, however the complex social conditions which surround each individual patient can as might be expected, be construed as a primary contributor to their health outcome. We acknowledge that these exposures investigated in this study are only a piece of the larger network of factors that impact OUD patients’ health outcomes.

Additionally, the cross sectional nature of this study is a factor that may compromise the quality of the evidence obtained. We are unable to establish a temporal relationship between the age of onset of opioid use and the presence of comorbid disorders. An inception cohort study design would be ideal to establish the age of onset of opioid use and ensuring all participants were free of physical and psychiatric comorbidity at baseline. However, following patients from the onset of opioid use onto the development of addiction may prove both unethical and unfeasible given the need for early intervention if addiction is suspected and the potential high attrition rate.

Finally, our comorbidity outcome was dichotomized which may take away from the magnitude of the burden of certain diseases. Now that we have established that an association does exist, we encourage future research to investigate the association between age of onset of opioid use and 10-year mortality risk or quality of life, using surrogate outcomes such as the Charlson Comorbidity Index [43].

## Conclusion

Our study identified a significant association between age of onset of opioid use and the presence of a physical or psychiatric comorbidity in patients receiving MMT for OUD. Specifically, a younger age of onset was found to be associated with an increased likelihood of having a physical and/or psychiatric comorbidity. The results of this study may serve to enhance therapeutic guidelines for patients receiving MMT by identifying high-risk patient groups and addressing them accordingly. The opportunity to identify these patients would allow us to monitor them more closely, provide more intensive treatment strategies as well as implement preventative measures to reduce the risk of them developing a disease and/or progressing in their course of illness.

## Abbreviations

MMT: methadone maintenance therapy
OUD: opioid use disorder
HIV: human immunodeficiency virus
GENOA: Genetics of Opioid Addiction
CATC: Canadian Addiction Treatment Center
DSM-5: diagnostic and statistical manual of mental disorders
M.I.N.I.: Mini-International Neuropsychiatric Interview
RedCap: Research Electronic Data Capture
